# Changed functional connectivity at rest in functional illiterates after extensive literacy training

**DOI:** 10.1186/s42466-020-00058-0

**Published:** 2020-05-12

**Authors:** Bahram Mohammadi, Thomas F. Münte, David M. Cole, Amir Sami, Melanie Boltzmann, Jascha Rüsseler

**Affiliations:** 1 CNS-LAB, International Neuroscience Institute (INI), Hannover, Germany; 2 Department of Neurology, University of Lübeck, Ratzeburger Allee 160, 23562 Lübeck, Germany; 3 Institute of Psychology II, University of Lübeck, Lübeck, Germany; 4 Translational Neuromodeling Unit, Institute for Biomedical Engineering, University of Zurich & ETH Zurich, Zurich, Switzerland; 5 Department of Psychology, University of Bamberg, Bamberg, Germany; 6 Bamberg Graduate School of Cognitive and Affective Sciences (BAGrACS), Bamberg, Germany; 7 Neurologische Klinik Hessisch Oldendorf, Hessisch Oldendorf, Germany

**Keywords:** Functional connectivity, Functional illiteracy, Independent component analysis, Training intervention

## Abstract

**Background:**

About 6.2 million adults in Germany cannot read and write properly despite attending school for several years. They are considered to be functional illiterates (FI). Since the ability to read and write is crucial for being employed and socially accepted, we developed a special literacy training to overcome these deficits.

**Methods:**

In this study, we investigate training-related changes in intrinsic functional connectivity (iFC) at rest in a group of 20 FI and 20 adult normal readers using resting state functional magnetic resonance imaging (rsfMRI). We used independent component analysis (ICA) to define different networks.

**Results:**

Before training, the between group analysis showed increased iFC in FI in a left-fronto-parietal network (LFPN; anterior insula, medial frontal cortex, lateral and frontal parietal regions) and in the Basal Ganglia network (BGN: thalamus, caudate, putamen, pallidum, amygdala, supplementary motor cortex and cingulate gyrus). Furthermore, the Visual Network-1 (VN1; temporal occipital fusiform gyrus, lateral occipital cortex, occipital pole, lingual gyrus, thalamus) showed decreased iFC in FI. After training the FI group showed reversal of the “hyperconnectivity” in middle frontal gyrus and in the frontal orbital cortex and between supramarginal gyrus and the BGN. Furthermore, functional connectivity increased in FI VN1 (lateral occipital cortex, insular cortex). These changes in connectivity correlated with gains in reading speed and spelling accuracy.

**Conclusions:**

These findings show that poor reading and writing abilities are associated with abnormalities in iFC in several brain areas subserving cognitive processes important for reading. Intensive literacy training induces changes in the functional connectivity between and within neural networks important for literacy skills.

## Introduction

While fluent reading is the default in developed countries and most children learn to read and write without major problems, reading and writing can be disturbed. Much research has been devoted to developmental dyslexia, an unexpected difficulty with accurate and fluent reading which occurs despite normal intelligence, motivation, and exposure to adequate reading instruction. Developmental dyslexia occurs in 5–17% of the children [68] and problems may persist into adulthood. Functionally illiterate (FI) adults show even greater difficulties during reading acquisition than dyslexic individuals. Although they received the same reading and writing instructions as typically developing readers, FI left school with literacy skills that are at least three to four years below the expected level of performance [23, 24, 30, 80]. Typically, FI are unable to read and understand even short sentences. Single word reading of frequently occurring words is sometimes uncompromised [24]. Recent surveys conclude that there are about 6.2 million FI in Germany (i.e. 12.1% of the adult population [34];), with similar prevalence rates in France (9% [2];), and the UK (16% [82];). Theories of functional illiteracy [22, 24] have focused on environmental factors in childhood concerning school (e.g. truancy, inappropriate instructions, repetition of classes) and the family environment (e.g. neglect, drug abuse of parents, abuse, numerous siblings etc.). Recent research suggests that these negative experiences do not apply for all individuals with low literacy skills, and are also not sufficient to let someone become functionally illiterate [24, 80]. Accordingly, some researchers proposed that FI results from cognitive deficits coupled with environmental disadvantages [10, 24, 29, 31]. Moreover, a relationship between functional illiteracy and developmental dyslexia is assumed. As an example, Greenberg et al. [31] consider functional illiteracy as an adult form of developmental dyslexia not adequately treated in childhood (see also [80]). Accordingly, several studies showed similarities between the cognitive profiles and neurobiological underpinnings of reading in FI and in dyslexic readers [24, 29, 31, 33, 64].

The ability to read is supported by several distributed networks of different brain regions. Reviewing mainly task-based fMRI-studies, Grigorenko [32] identified three important brain circuits: A frontal system (inferior frontal gyrus), a temporo-parietal system (angular and supramarginal gyri, posterior part of the superior temporal gyrus [STG]), and an occipito-temporal system (occipito-temporal area and posterior parts of the middle and inferior temporal gyri). It has been shown that the frontal and the temporo-parietal systems are involved in phonological processing [32] and in grapheme-to-phoneme conversion. The occipito-temporal system is involved in “whole-word” recognition of word forms [69].

Resting-state fMRI (rsfMRI) can be used to study intrinsic functional connectivity (iFC) of the brain. It is detected by examining inter-regional correlations in spontaneous low-frequency fluctuations (< 0.1 Hz) in the rsfMRI-signal [8]. Koyama et al. [47] used rsfMRI to investigate iFC of the reading networks at rest. The authors chose six seeds roughly corresponding to the three reading systems described previously by Grigorenko [32] and in meta-analyses by Turkeltaub et al. [79] and Bolger et al. [9]: left gyrus fusiformis, left superior temporal gyrus, left temporo-parietal junction, left precentral gyrus, left inferior frontal gyrus, and left inferior occipital gyrus. The observed connectivity patterns largely overlapped with findings from task-based fMRI-studies during word reading. Furthermore, negative iFC was reported for several regions involved in effortful and controlled processing (i.e. dorsolateral prefrontal and superior parietal areas) as well as in the default mode network. The posterior left inferior frontal gyrus (post-LIFG) and the posterior left middle temporal gyrus (post-LMTG) were identified as possible loci of functional integration among the reading networks. These findings demonstrate the usefulness of rsfMRI to study the functional organization of reading in the brain. It is important to note, however, that many studies have demonstrated that reading engages many regions beyond the aforementioned reading networks and in particular limbic and subcortical regions [1, 3, 41, 53, 54].

A handful of studies have used rsfMRI to investigate the differences in iFC in reading networks of dyslexic and typically reading children [86] and/or explored changes in iFC of reading-related brain networks related to remediation [42, 43, 48]. Dyslexics differed from typical readers in iFC between the intraparietal sulcus and the visual word-form area as well as in the connections of these structures to the left-middle frontal gyrus [86]. Schurz et al. [66] observed reduced task-related and resting-state connectivity for dyslexics between seeds in left posterior temporal areas and the inferior frontal gyrus. This implies that the disruption of functional integration between frontal and temporal brain areas in dyslexic readers is a permanent one as it is present during task performance and in the absence of a task. Exploring the effects of reading remediation on iFC, Koyama et al. [48] studied (1) dyslexic children with current reading and spelling difficulties, (2) dyslexic children that received training and had only reading difficulties at test (partly remediated group), (3) fully remediated, i.e. no more reading and spelling deficits, (4) non-dyslexic typical readers. Before training, less iFC was observed for dyslexics between two seeds in the left posterior reading network (intraparietal sulcus [L.IPS] and left fusiform gyrus [L.FFG]). After remediation, all dyslexic groups showed weaker iFC between L.IPS and the left middle frontal gyrus. Both remediation groups showed stronger iFC between the L.FFG and right middle occipital gyrus compared to typical readers and non-remediated dyslexics after training. Furthermore, the full remediation group showed stronger negative iFC between L.FFG and the right medial prefrontal cortex. This pattern of brain connectivity at rest suggests that successful remediation of dyslexic children is associated with the emergence of compensatory changes in L.FFG iFC. These studies demonstrate the usefulness of rsfMRI in the analysis of training-related changes in iFC in the brain networks involved in reading.

While the previous studies in dyslexia focused on reading skills, it must be emphasized that dyslexic children and adults also suffer from writing problems [28, 36, 37]. This is true to a greater extent in FI. To date, only one study used resting state with functional illiterates: Skeide et al. [71] observed literacy-induced neural plasticity in adult Indian illiterates. Six months of reading instruction lead to increases in the functional connectivity between the occipital lobe and subcortical areas (in the midbrain and thalamus) that were related to individual gains in reading ability.

The present study aimed at exploring changes in iFC at rest associated with reading training in a group of FI adults, thus adding to the previous study of Skeide et al. [71] drawing on subjects raised in an industrialized country. Furthermore, pre-training differences in iFC at rest between the group of FI readers and normal readers were investigated. Twenty adult FI were scanned before and after a 7 month long training of reading and writing abilities (AlphaPlus, [10]). On average, pre-training level of reading was comparable with that of a first-grade student. After training, reading was comparable to a third grade student. Twenty adult normal readers served as a control group.

We used a data-driven ICA-approach to identify resting-state networks. In a second step, we looked for differences in functional connectivity between the two groups before training. For the group of FI, we also looked at the effect of training on functional connectivity. To the extent to which findings in dyslexic readers [42, 43, 66, 86] also apply to FI, we hypothesized decreased functional connectivity in FI in comparison to a group of typical readers in reading-related and non-reading-related neural networks. We also hypothesized that literacy training would normalize functional connectivity in the very same networks that show differences before training (c.f., [48], for a similar study in dyslexia). Specifically, following Skeide et al. [71] literacy training was hypothesized to increase FC between the occipital lobe and subcortical areas.

## Methods

### Participants

Twenty adult FI participated in the study (15 men, mean age 42.70 years, *SE* = 2.09 years; range 25 to 58 years, 16 right-handed). They were recruited from an adult literacy program (AlphaPlus, [11, 65]) and received daily training over a period of 7 months using a formal literacy instruction approach combined with computer-based exercises. Although the participants had attended school for several years (*M* = 8.95 ± 0.37 years), they demonstrated poor literacy skills (see below) and were considered to be functionally illiterate.

The control group comprised 20 adults (15 men, mean age 44.93 years, *SE* = 3.57 years; range 27 to 56 years, 16 right-handed). Individuals were included if their reading and writing skills were within a range that can be expected for their age and formal educational status. It was also made sure that they had no former diagnosis of any reading, writing or general learning impairment in childhood.

General inclusion criteria for both groups were: (1) German as the primary language, (2) age above 18 years, (3) general cognitive ability in the normal range and (4) normal hearing and vision. Functional illiterates and control subjects were individually matched for age (*T* (39) = 0.72, *p* = 0.47) and gender (*Χ*^*2*^(1) = 0, *p* = 1). The study was conducted in accordance with the declaration of Helsinki. Furthermore, the study protocol was approved by the ethical committee of the University of Bamberg. All participants gave their written informed consent and were paid for test participation (10 € per hour).

### Assessment of reading and writing skills

Reading abilities of functionally illiterate adults were assessed with a standardized German reading test (Würzburger Leise-Leseprobe, WLLP [50]). In this test, 140 written words as well as four pictures next to each word are presented. The participants have to mark the one picture that represents the word on the left side. The test score comprises the number of correctly identified pictures in 5 min. The WLLP is supposed to measure silent reading speed and the ability to decode written words. Writing abilities of functionally illiterate adults were tested with a standardized German writing test for first graders (Diagnostischer Rechtschreibtest, DRT-1 [58]). Here, participants have to write 32 single words from dictation. We used parallel versions of the WLLP and DRT-1 at the beginning as well as at the end of the training in order to assess the efficacy of the training program.

The functional illiterates were scanned prior to and after they participated in a 7 month long reading instruction program (AlphaPlus; see above and [11, 65]).

### Literacy training program

The literacy training program AlphaPlus consists of seven modules that address different aspects of reading and spelling skills. In the present study, course duration was 7 months Monday to Friday. Literacy training duration was approximately 2 to 3 h daily. Module 1 (classical literacy lessons) consists of four modules with increasing complexity which can be used flexibly in the classroom. For each module, there is a textbook containing specific tasks. These are available online at https://www.bnw.de/bnwde/content/deutsch/unternehmen/qualifizierung/grundbildung. The audio trainer consists of eight tasks that are designed to train basic perceptual functions in the visual, auditory and motor domain. These perceptual abilities are trained at the beginning of the program; later, only refresher sessions take place. With the help of the Alpha-Trainer (module 3), language and perceptual functions are promoted through “lateralized synchronous speech”. The trainee vocalizes individual syllables, words, sentences or texts in sync with a model voice. The two different language information (model voice and own voice) are constantly moving from one ear to another in opposite directions (via headphones). In this way, the two voices can be perceived separately and can be compared with each other. In addition, the training elements are presented acoustically as well as visually fostering audio-visual integration as well as the coordination of both brain hemispheres. The training is based on findings suggesting that the corpus callosum is smaller and functionally impaired in individuals with literacy problems [26]. The corpus callosum has been implicated, among other things, in sensory integration processes and in the transfer of information between the hemispheres [62]. The fourth module, the sound discrimination trainer, fosters the distinction between consonants and vowels. The trainee hears short words presented alternately to the left and right ear. Each stimulus begins and ends with a vowel; the middle letter is an alternating consonant (e.g. “eki”, “efi”) or a letter combination (e.g. “ch”, “sch”). By pressing a button, the trainee specifies the perceived middle sound. Module 5 comprises computer-based spelling training using Orthofix®. On a computer screen, a word is presented, which is either spelled out or read by a model voice. The word disappears after a few seconds and the trainee is prompted to rewrite the word with the keyboard. In simple training mode, word typing is only practiced forward. In the advanced mode, the displayed words have to be typed forward and backward. In its basic form, Orthofix® contains about 10.000 of the most common German words, sorted by topic. In addition, there are job-specific word lists that allow the parallel learning of job-specific terminology. Currently, job-relevant words are implemented for 15 different occupational groups (e. g., professional care, roofers, butchers, landscaping, scaffolders). It is also possible to create own word lists. Another component of the training (module 6) is the online learning platform www.ich-will-lernen.de of the German Adult Education Association. This is Germany’s largest literacy learning platform with more than 31.000 different exercises. Course participants worked through individually chosen exercises dependent on their reading and writing level. Module 7 consists of various social training exercises like fitness training, joint cooking, communication training and participation in cultural events.

### Image acquisition

Magnetic-resonance images were acquired on a 3-T Siemens Scanner (Erlangen, Germany) equipped with a standard head coil. A total of 178 T2*-weighted volumes of the whole brain (EPI-sequence; TR 2000 ms, TE 30 ms, flip angle 80°, FOV 192 mm, matrix 64 × 64, 34 slices, slice thickness 3 mm, interslice gap 0.75 mm) parallel to the anterior-posterior commissural line were recorded for functional imaging resulting in a 6 min run, a duration that has been commonly used in rsfMRI [27].

A T1-weighted high resolution data set was acquired using a 3D-MPRAGE (3 dimensional magnetization prepared rapid acquisition gradient echo) sequence for anatomical information (matrix 192 × 256, 1 mm isovoxel). The subject’s head was fixed during the entire measurement to avoid head movements.

### FMRI data analysis

FMRI analysis at rest was carried out using Multivariate Exploratory Linear Optimized Decomposition into Independent Components (MELODIC) Version 3.14 part of FSL (FMRIB’s Software Library, www.fmrib.ox.ac.uk/fsl) [4].

Preprocessing consisted of motion correction, brain extraction, spatial smoothing using a Gaussian kernel of full-width at half maximum (FWHM) of 6 mm, and high-pass temporal filtering equivalent to a time-constant of 150 s (0.007 Hz). For motion detection and correction, six realignment parameters, i.e. three displacements and three elementary rotations, were obtained with respect to the first image in the EPI series. The displacements with respect to the first image of the series were required to be smaller than 3.0 mm (minimum to maximum) and the individual rotations were required to be smaller than 3.0°. None of the participants had to be excluded according to these criteria. FMRI volumes were registered to the individual’s structural scan and standard space images using FMRIB’s Nonlinear Image Registration Tool (FNIRT). Preprocessed functional data containing 178 time points for each subject were temporally concatenated across subjects to create a single 4D data set. The between-subject analysis of the resting data was carried out using a regression technique (dual regression) that allows for voxel-wise comparisons of resting functional connectivity [25]. This approach proceeds in 3 stages. First, the concatenated multiple FMRI data sets are decomposed using ICA to identify large-scale patterns of functional connectivity in the population of subjects. In this analysis, the data set was decomposed into 30 components, in which the model order was estimated using the Laplace approximation to the Bayesian evidence for a probabilistic principal component model [5].

Second, the dual-regression approach is used to identify, within each subject’s FMRI data set, subject-specific temporal dynamics and associated spatial maps. This involves first using the full set of group-ICA spatial maps in a linear model fit (spatial regression) against the separate fMRI data sets, resulting in matrices describing temporal dynamics for each component and subject, then using these time-course matrices in a linear model fit (temporal regression) against the associated fMRI data set to estimate subject-specific spatial maps.

These spatial maps characterize the subject- and voxel-specific degree of integration into a given group component map. Finally, the different component maps are collected across subjects into single 4D files (1 per original ICA map, with the fourth dimension being subject identification) and tested voxel-wise for statistically significant differences between groups using nonparametric permutation testing [59]. This results in spatial maps characterizing the between-subject/group differences. These maps were thresholded at *p* < 0.05 (Family-wise error corrected) using Threshold-free cluster enhancement (TFCE [73]) to define clusters of significant changes in connectivity. Data were visualized in MNI standard space using FSLView.

### Seed based correlation analysis (SBCA)

Subject/session-specific measures of voxel-wise subcortical connectivity with each of the RSNs were obtained using Seed Based Correlation Analysis; SBCA tool part of FSL [15, 60], for controls and functional illiterates before and after treatment separately. SBCA was carried out within a subject-specific, anatomically-derived subcortical seed mask. Every voxel within each ‘individualized’ seed mask was quantitatively tested in terms of its connectivity with each of the RSN target maps. We employed two variations on the analysis approach reported previously in a study of healthy volunteers [15], both of which were incorporated to account for the potential (but relatively unclear) contribution of tissue macro- or micro-structural variations (e.g., atrophy, cortical thinning) to regional neuroanatomical changes. Firstly, to ensure more accurate alignment of neural systems defined at a group level to the brain space of individuals, prior to affine transformation to EPI space, RSN target maps from group-ICA were nonlinearly (as opposed to linearly) transformed from MNI space to the high-resolution space of each subject (as implemented in FSL FNIRT); wherein, secondly and prior to registration to EPI space, voxels failing to exceed a comparatively liberal threshold of > 10% probability of containing grey matter (relative to > 20% applied previously, as calculated using FSL FAST) in the equivalent T1 structural were removed from these ‘subject-specific’ RSN spatial maps.

To construct subject-specific subcortical seed masks, T1 structural images were segmented using FSL FIRST. Bilateral regions included in these masks were the entire striatum (comprising regions of caudate, putamen and ventral striatum), pallidum, amygdala, hippocampus and thalamus (and midbrain, see below). The unthresholded versions of these segmented structures (i.e., without boundary correction; see [61] were combined into a single mask image for each subject. To include midbrain voxels within our masks, we carried out nonlinear warp transformation (using FNIRT) of six binary, bilateral volumes (midbrain, substantia nigra, subthalamic nucleus, red nucleus, mammillary body and medial geniculum body) from the Talairach Daemon atlas [52] to the high-resolution space of each subject. This midbrain information was then added to the mask containing subjects’ other subcortical regions. These subject-specific combined masks were then affine registered to EPI space using FSL FLIRT and used in subsequent subject-wise SBCA, to quantify subcortical functional connectivity with large-scale RSNs.

### First-level analysis

In SBCA the subcortical seed masks from each subject were examined individually, in EPI space, for their voxel-wise spatial distributions of functional connectivity strength with the characteristic activity of each of the RSNs. Voxel-wise connectivity strengths were quantified by calculating partial correlation coefficients between the BOLD signal time series at each mask voxel and that of the weighted principal eigenvariate associated with each RSN (the latter calculated via subject-wise principal component analyses [60]. Voxel-wise coefficients are termed ‘partial’ because the analysis associated with a given target RSN controlled, in turn, for the seed voxel’s activity relationship with each of the other RSNs examined as targets in separate correlation analyses. In these analyses we also controlled for the confounding influences of structured noise from white matter (WM) and cerebrospinal fluid (CSF) tissue types and residual motion artifacts. To this end, binary T1-segmented maps of WM and CSF (calculated using FAST) were registered to EPI space using FLIRT and, for each session, used as masks against the associated, preprocessed functional datasets, in order to extract confound time series that were calculated as the mean BOLD signal within these tissue masks. As well as these WM and CSF confounds, six time series resulting from the motion correction procedure (as implemented in FSL MCFLIRT) describing individual subject head motion parameters were also regressed out of the SBCA. Paired and unpaired t-tests revealed that mean and relative head motion did not differ significantly between training sessions or subject groups (all *p* > 0.5).

### Higher-level analyses

We used all RSNs in higher-level analyses. The partial connectivity between subcortical seed masks and each target was tested voxel-wise for statistically significant differences between groups using nonparametric permutation testing [59]. This results in spatial maps characterizing the between-subject/group differences. These maps were thresholded at *p* < 0.05 (FWE corrected) using TFCE [73] to define clusters of significant changes in connectivity. Statistical contrasts in the higher-level analysis specifically examined RSN functional connectivity patterns for differences between controls and functional illiterates and basic paired effects of treatment.

To investigate interactions between training-dependent neurobiological effects and psychological scores revealed in this study, bivariate correlations (Pearson’s r, *p* < 0.05, two-tailed) were carried out between individual subject measures of functional connectivity in different networks and psychological parameters. Significant clusters identified from the difference between pre- and post-training were thus used as masks to extract mean values from individual data sets. The differences in these values resulting from training and that of psychometric parameters were used to test for correlation.

## Results

### Reading and writing skills

Literacy scores for both groups are presented in Table 1. With regard to reading, the FI group showed significant improvement due to the training (t (19) = 3.78, *p* < 0.01), but still did not reach the reading level of the control participants after training (t (30) = 8.00, *p* < 0.001). Likewise, the number of writing errors significantly decreased in the FI group due to the training (t (19) = 6.25, *p* < 0.001). Again, their error level after training was considerably higher than that of the control group (t (30) = 4.14, *p* < 0.001).

**Table 1:** Literacy skills of functional illiterates and controls (mean and SD)

	Functional illiterates (*n* = 20)		Controls (*n* = 20)
Reading skill (correct words)	before training	39.30 (5.85)	135.25 (7.66)
	after training	55.20 (7.66)	
Writing skill (errors)	before training	16.10 (2.36)	0.00 (0.00)
	after training	10.25 (1.91)	

### Independent component analysis

The ICA decomposition resulted in 30 spatial maps, containing classically identified resting-state networks [4, 19, 21] as well as artefactual components. The complete set of 30 components was used for the dual-regression analysis. Only three of the original components showed between-group changes in the degree of functional connectivity: the left Fronto-Parietal Network (LFPN), the Visual Network (VN) and the Basal Ganglia Network (BGN).

#### Left Fronto-parietal network (LFPN)

**Fig. 1:**
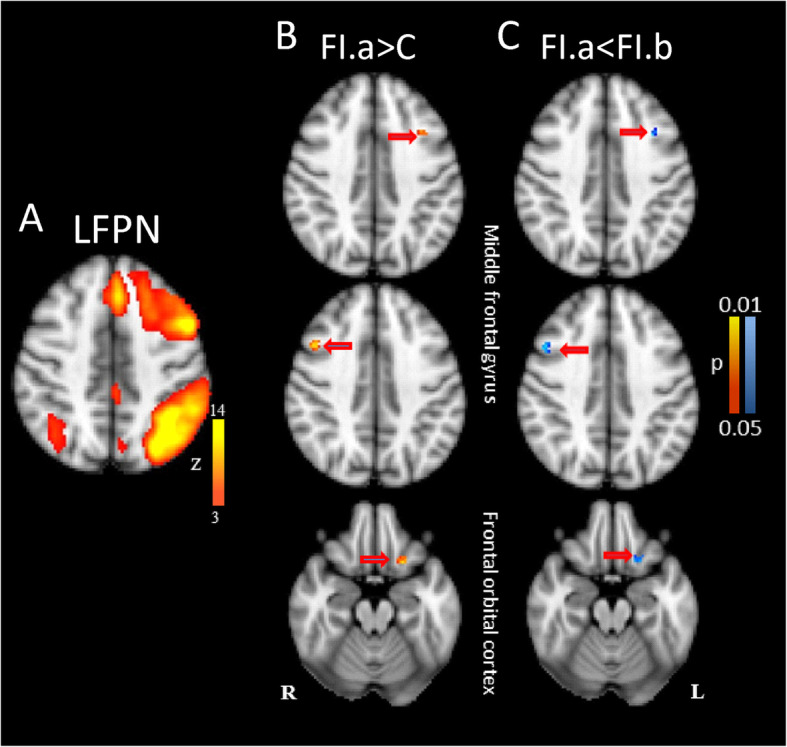
**a** left Fronto-Parietal Network (LFPN) as revealed by ICA. **b** Between group comparison in LFPN comparing functional illiterates before training (FI.a) and control group (C). **b** Training induced changes in LFPN in the FI group: Training led to a normalization. All data are FWE-corrected for multiple comparisons (*p* < 0.05)

Two lateralized frontoparietal networks (left: LFPN, right: RFPN) were identified, each comprising anterior insula, medial frontal cortex, and lateral frontal and parietal regions. Only the LFPN showed increased connectivity in FI in middle frontal gyrus and frontal orbital cortex compared to the control group (Fig. 1; Table 2).

**Table 2:** Regions with changed functional connectivity in functional illiterates (FI) before training (*n* = 20) compared to controls in different networks

Brain region	Hemisphere	MNI coordinates			Cluster size	*p* (FWE)
		X	Y	Z		
**Controls < FI before training**						
**LFPN**						
Middle Frontal Gyrus	L	−38	14	40	24	0.03
Middle Frontal Gyrus	R	46	16	40	55	0.02
Frontal Orbital Cortex	L	−22	20	−22	30	0.02
**BG Network**						
Supramarginal Gyrus, posterior division	R	56	−40	26	24	0.03
**Controls > FI before training**						
**Visual Network**						
Lateral Occipital Cortex, inferior division	L	−36	−78	−4	26	0.03
Insular Cortex	L	−34	14	−12	84	0.02

#### Basal ganglia network (BGN)

**Fig. 2:**
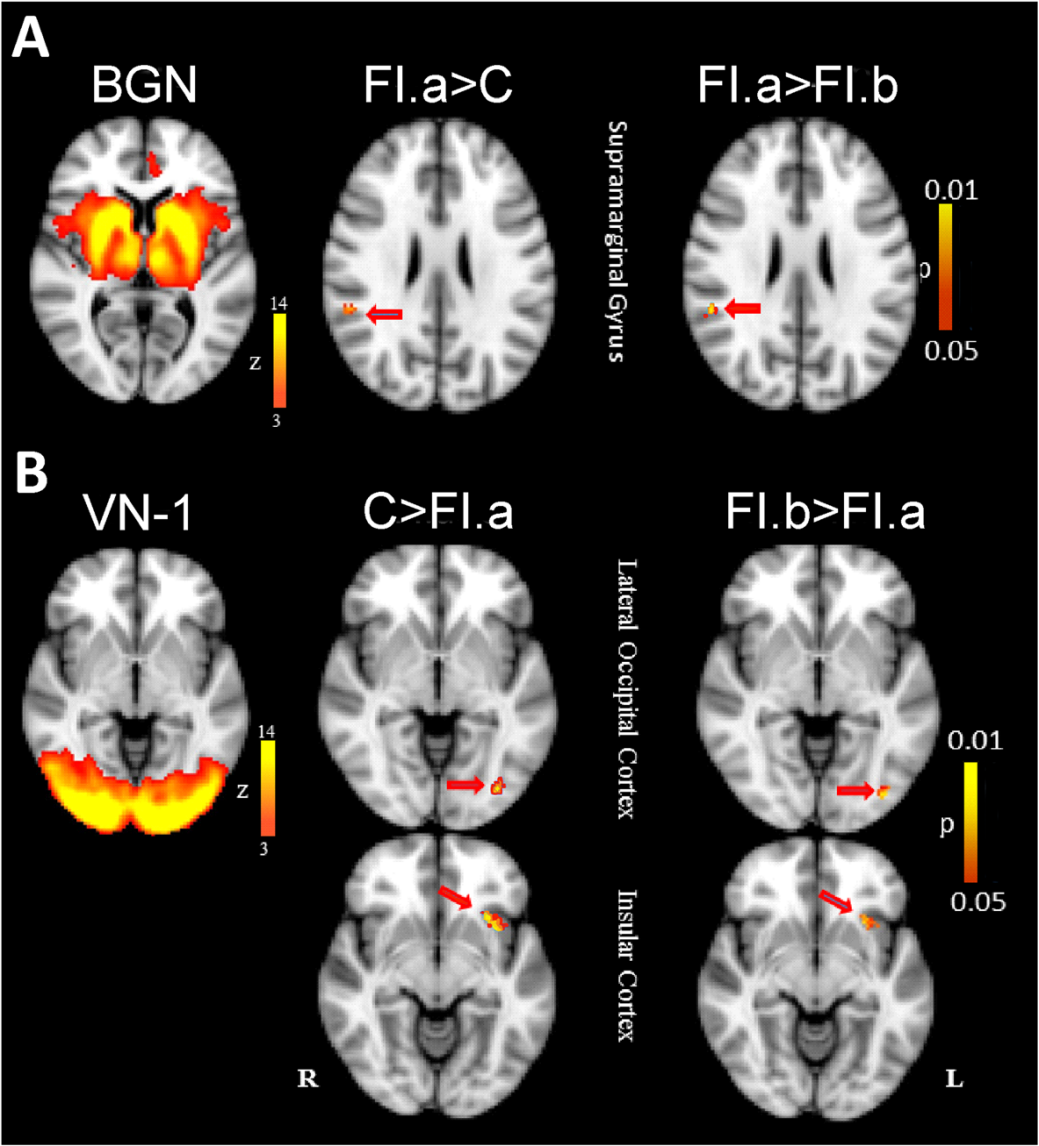
**a** basal ganglia network (BGN, left); between group comparison comparing FI before training (FI.a) and control group (C) (middle); training effect comparing FI before (FI.a) and after (FI.b) training (right). **b** Visual Network-I (VN-1, left column); between group comparison comparing FI before training (FI.a) and control group (C) (middle); training effect comparing FI before (FI.a) and after (FI.b) training (right). All data are FWE-corrected for multiple comparisons (*p* < 0.05)

This network included thalamus, caudate, putamen, pallidum, amygdala, insular cortex, central opercular cortex, supplementary motor cortex and cingulate gyrus. The group analysis showed increased functional connectivity between right supramarginal gyrus, posterior division and this network in FI (Fig. 2a; Table 2).

#### Visual networks (VN)

ICA identified two visual cortical sub-networks: the first consisting of temporal occipital fusiform gyrus, lateral occipital cortex, occipital pole, lingual gyrus and thalamus (VN1); and the second comprising intracalcarine cortex, lingual gyrus, occipital pole, occipital fusiform gyrus and cuneal cortex (VN2). We found a significantly decreased functional connectivity of left lateral occipital cortex in VN1 in FI and also decreased connectivity of left insular cortex to this network in same group (Fig. 2b; Table 2). The between group analysis showed no significant changes in VN2.

### SBCA

At this step we focused our analysis on functional connectivity between different subcortical areas (including the entire striatum comprising the caudate nucleus, putamen and ventral striatum, pallidum, amygdala, hippocampus, thalamus and midbrain) and neocortical networks found in this study. The between group analysis showed changes of connectivity between distinct subcortical areas and neocortical networks including default mode network (DMN), dorsal attention network (DAN), ventral attention network (VAN) and LFPN.

**Fig. 3:**
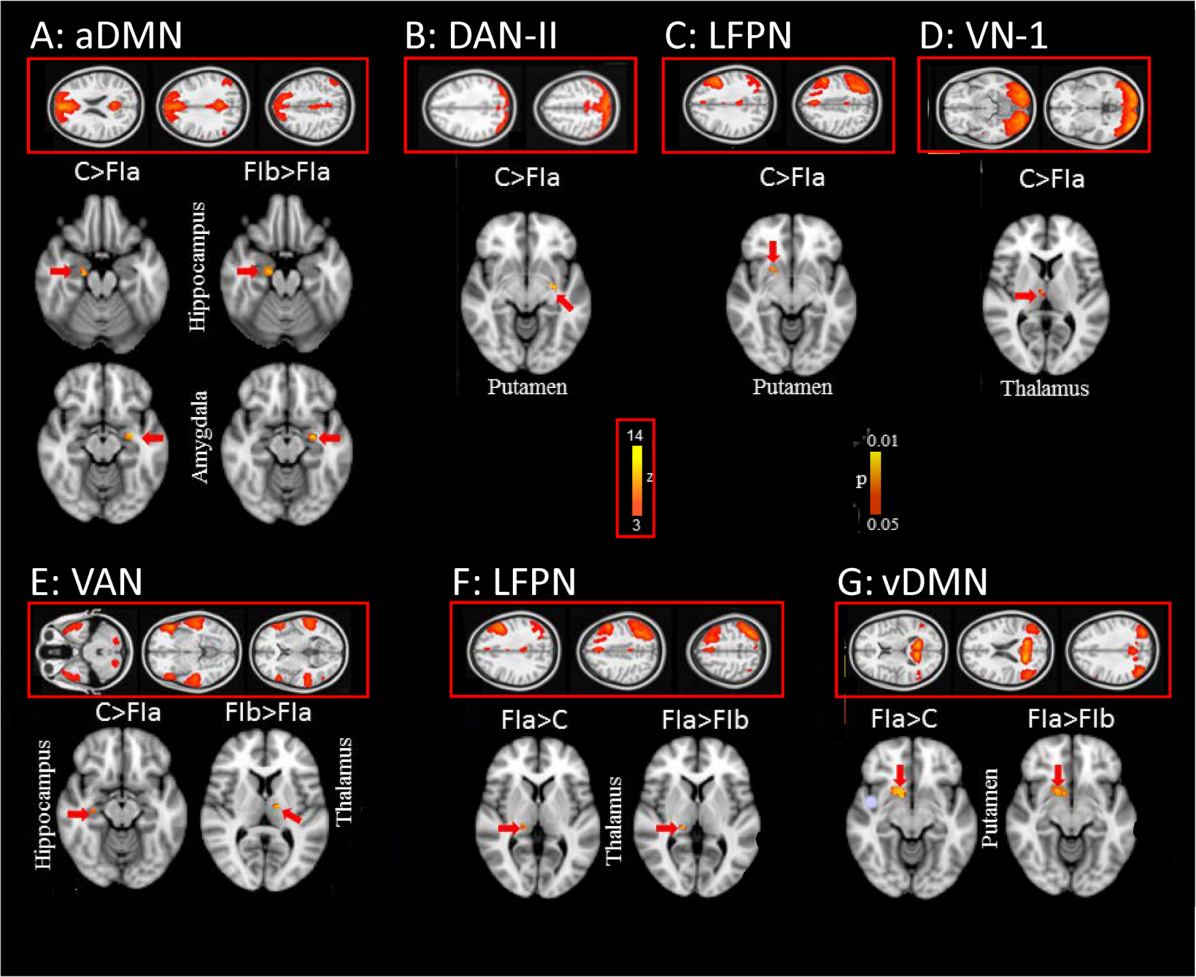
Seed Based Correlation Analysis (SBCA) between selected subcortical areas (indicated by red arrows) and different neocortical networks. **a** anterior default mode network (aDMN); **b** dorsal attention network II (DAN-II); **c**/**f** left fronto-parietal network (LFPN); **d** visual network-I (VN-1); **e** ventral attention network; **g** ventral default mode network (vDMN). All data are FWE-corrected for multiple comparisons (*p* < 0.05). C: Control group; FI.a: functional illiterates before training; FI.b: functional illiterates after training

The DMN comprised prefrontal, anterior and posterior cingulate, lateral parietal, and inferior/middle temporal gyri (extending to the mesial temporal lobe), cerebellar areas, and thalamic nuclei. The ICA divided this network in three sub networks including anterior DMN, ventral DMN and posterior DMN. The between group analysis showed reduced connectivity between anterior DMN and left amygdala/right hippocampus in FI (Fig. 3a, Table 3). However, the connectivity was increased between ventral DMN and a cluster spanning right putamen and right Ncl. accumbens in the same group (Fig. 3g, Table 3).

**Table 3:** Changed connectivity between different subcortical and neocortical networks compared between Controls and functional illiterates (FI) before training

Brain region	Hemisphere	IC/Network	MNI coordinates			Cluster size	*p* (FWE)
			X	Y	Z		
**Controls > FI before training**							
Hippocampus	R	anterior DMN	20	−12	20	53	0.02
Amygdala	L	anterior DMN	−28	−8	−16	30	0.01
Putamen	L	DAN (Precuneus)	−30	−10	−6	27	0.01
Putamen	R	LFPN	20	10	−8	19	0.01
Thalamus	R	Visual-II	6	−14	6	16	0.03
Hippocampus	R	VAN	30	−20	−14	95	0.01
**FI before training > Controls**							
Thalamus	R	LFPN	16	−28	4	15	0.02
Putamen	R	ventral DMN	18	10	−10	98	0.01
Accumbens	R	ventral DMN	10	10	−8	47	0.01

The VAN consisted of precuneus cortex, cingulate gyrus, angular gyrus, superior frontal gyrus and middle frontal gyrus and showed reduced connectivity to the right hippocampus (Fig. 3e, Table 3) in FI, relative to controls. The DAN was divided into two subnetworks; DAN-I comprising lateral occipital cortex, superior parietal lobule, supramarginal gyrus, inferior temporal gyrus, superior frontal gyrus, inferior frontal gyrus, precentral gyrus and postcentral gyrus and DAN-II with extension to dorsal precuneus. We found significantly reduced connectivity between DAN-II and the left putamen (Fig. 3b, Table 3) in FI, relative to controls.

Furthermore, we found reduced connectivity between VN1 and right thalamus (Fig. 3d, Table 3) and between LFPN right putamen (Fig. 3c, Table 3) in FI but increased connectivity of LFPN to right thalamus (Fig. 3f, Table 3).

### Training effects

The gain in reading and writing abilities in FI was associated with the following neural changes: In LFPN the training resulted in the reversal of ‘hyperconnectivity’ (relative to control subjects) in FI in middle frontal gyrus and frontal orbital cortex (Fig. 1, Table 4). The hyperconnectivity between supramarginal gyrus and BGN in FI was reversed after training (Fig. 2a, Table 4). Considering VN1 we found increased functional connectivity in lateral occipital cortex and insular cortex after training, reaching the same level as in healthy controls (Fig. 2b, Table 4).

**Table 4:** Regions with changed functional connectivity in functional illiterates after training (*n* = 20) compared to before training in different networks

Brain region	Hemisphere	MNI coordinates			Cluster size	*p* (FWE)
		X	Y	Z		
**After < Before training**						
**LFPN**						
Middle Frontal Gyrus	L	−36	14	40	10	0.03
Middle Frontal Gyrus	R	44	14	42	23	0.03
Frontal Orbital Cortex	L	−22	22	−22	26	0.02
**BG Network**						
Supramarginal Gyrus, posterior division	R	56	−40	24	17	0.03
**After > Before training**						
**Visual Network**						
Lateral Occipital Cortex, inferior division	L	−36	−82	−4	12	0.03
Insular Cortex	L	−32	16	−10	53	0.03

The connectivity between subcortical areas and different networks in FI shown above was also influenced by training (Fig. 3, Table 5): training increased the connectivity between anterior DMN and left amygdala/right hippocampus and between VAN and left thalamus. However, we found a significant decrease of connectivity between LFPN and right thalamus and between ventral DMN and right putamen/right Ncl. accumbens.

**Table 5:** Subcortical areas with significant changes of connectivity to different networks in functional illiterates after training

Brain region	Hemisphere	Network	MNI coordinates			Cluster size	*p* (FWE)
			X	Y	Z		
**After training > before training**							
Hippocampus	R	anterior DMN	22	−16	−20	67	0.02
Amygdala	L	anterior DMN	−26	−8	−16	18	0.01
Thalamus	L	VAN	−10	−12	8	74	0.01
**Before training > after training**							
Thalamus	R	LFPN	16	−28	2	14	0.03
Putamen	R	ventral DMN	18	10	−10	62	0.01
Accumbens	R	ventral DMN	10	10	−10	28	0.02

We also performed a comparison between the imaging data of controls and illiterates after literacy training. These analyses did not reveal significant effects at the chosen statistical threshold. Decreasing the statistical threshold revealed a pattern similar to the comparison between controls and illiterates before training.

### Correlation to reading and writing performance

The reversal of hyperconnectivity (i.e. the connectivity difference between pre- and posttraining sessions) of right middle frontal gyrus to the LFPN and of right supramarginal gyrus to the BGN correlated positively with reading ability as assessed by WLLP and negatively with the number of writing errors as assessed by DRT-1. The increase of functional connectivity in left insular cortex in VN1 also correlated positively with WLLP and negatively with writing errors (Table 6).

**Table 6:** Regions with changed functional connectivity in functional illiterates after training (*n* = 20) compared to before training in which we found significant correlation (*p* < 0.005, two tailed) to WLLP and DRT1. The table shows the Pearson coefficient (r)

Brain region	Hemisphere	Network	Reading (WLLP)	Writing (DRT-1)
Middle Frontal Gyrus	R	LFPN	0.48	−0.47
Insular Cortex	L	Visual	0.49	−0.45
Supramarginal Gyrus, posterior division	R	BG	0.46	−0.44

With regard to SBCA we found that increased connectivity between left amygdala and anterior DMN in FI after training correlated positively with WLLP (Pearson coefficient 0.68, *p* < 0.001 two tailed). We found no other correlations considering WLLP or DRT-1.

## Discussion

In this study we used rs-fMRI to elucidate differences in intrinsic functional connectivity between FI and normal readers and their response to intensive literacy training. Training led to a significant improvement of reading and writing skills. In line with our hypotheses, the differences in reading between normal readers and the FI group as well as the training-induced changes in reading performance were accompanied by changes of intrinsic connectivity of the brain *at rest*, i.e. when the participants were not required to read. In other words, the degree of reading ability leaves its traces in brain connectivity during the idling state of the brain.

To summarize, the between group analysis showed increased iFC in FI in the LFPN (anterior insula, medial frontal cortex, lateral and frontal parietal regions) and in the BGN (thalamus, caudate, putamen, pallidum, amygdala, supplementary motor cortex and cingulate gyrus) before training. Furthermore, VN1 (encompassing temporal occipital fusiform gyrus, lateral occipital cortex, occipital pole, lingual gyrus, thalamus) showed decreased iFC in FI.

Functional connectivity between the anterior component of the DMN and left amygdala / right hippocampus, between the DAN and the left putamen, between VAN and the right hippocampus as well as between VN1 and the right thalamus was reduced in FI. An increase in iFC in FI was found between ventral DMN and right putamen/right Ncl. accumbens and between LFPN and the right thalamus. Training-related changes of functional connectivity in the FI group comprised of reversal of the “hyperconnectivity” in middle frontal gyrus and in the frontoorbital cortex and between supramarginal gyrus and the BGN. Furthermore, functional connectivity increased in FI in the VN1 (lateral occipital cortex, insular cortex). These changes in connectivity correlated with gains in reading speed and spelling accuracy.

These findings add to the growing evidence that on a neural level, problems of reading and writing are reflected in differences in brain connectivity mainly involving the neural networks that support the reading process (e.g., [56, 83, 85]). Furthermore, learning to read and write in adulthood leads to changes in iFC in several brain regions relevant for reading (see also [71]).

It is important to emphasize again, that the current analysis is based on resting state fMRI recordings. Any differences between literate participants and FI participants may thus be either driven by the inability of the FI participants to read and / or by compensatory mechanisms engaged in the FI participants. For example, the inability to read my cause FI participants to rely on memory processes to a greater extent. Therefore, the fact that we found normalization of the connectivity differences due to training is very important and suggests that the differences between FI and typical reading participants are mainly due to their inability to read.

### Pre-training group differences in iFC in resting-state neural networks

The FPN is involved in rule-based problem solving and goal-directed behavior using and manipulating working memory [46, 55, 57, 63]. This network is thought to support online task-control that allows rapid adaptation of control settings from one event to the next. It is referred to as “task positive” because it increases in activity when attention is directed to external stimuli in cognitive tasks [75, 81]. Two lateralized FPNs with predominantly left (LFPN) and predominantly right (RFPN) brain areas involved can be distinguished. Both networks also comprise minor contributions from brain areas in the contralateral hemisphere (c.f [49].), as was also case in the current data set. According to a meta-analysis of the BrainMap database, RFPN activity associates predominantly with action inhibition, cognition and memory. However, in addition to cognitive memory functions the LFPN has also been found to associate significantly with processes subserving language-related cognition [72]. For example, this network is activated during tasks involving words, pseudowords, letters, or Asian characters [51]. The increased functional connectivity of LFPN in the FI group may reflect compensatory hyperconnectivity of this network involved in online task control and different aspects of language in untrained subjects. Alternatively, this hyperconnectivity may also reflect disease-related inhibition between networks. In any case, hyperconnectivity was normalized after the training, as indicated by the fact that the comparison between controls and illiterates after training did not reveal significant effects for the network. In line with these findings Horowitz-Kraus et al. [42, 43] reported training-dependent changes in attentional and cognitive control networks encompassing the middle frontal gyrus in a sample of dyslexic children aged 8–12 years.

The Visual Networks include areas that are known to be central to different aspects of neural processing within the visual hierarchy. The Visual Network-I in this study corresponds spatially to a network identified in BrainMap meta-analyses that additionally associates with language and orthography, which may reflect the perception of forms in written language and reading [51, 72]. In untrained FI we found Visual Network-I to display decreased connectivity with lateral occipital cortex and also with insular cortex. Both of these regions are also involved in language processing and may show decreased connectivity when under-utilized. Insula is supposed to be strongly involved in perception and awareness. Different task-related studies show increased activity in the insula as a component of various large-scale networks across a variety of cognitive tasks (for review see [76];). Decreased functional connectivity between insula and visual network in this study may mirror reduced awareness/perception of written language as meaningful visual stimuli in untrained FI [12]. This was reversed by training and the post-training increase of functional connectivity between left insular cortex and this network correlated positively with WLLP and negatively with DRT-1, which suggests increased awareness/perception of written language.

Besides its involvement in motor tasks several studies have shown that the basal ganglia network is involved in various reading and language tasks [13, 51, 78]. The results of BrainMap meta-analyses reveal functional networks activated during different tasks from more than 16,000 fMRI scans. These task-dependent networks strongly correspond to resting state networks, such as those found in this study. It is thus supposed that the same areas activated in a network during a task remain highly functionally connected with each other at rest [72].

### Group differences in functional connectivity between different subcortical areas

At first glance, the clear implication of subcortical regions in the current analysis may seem somewhat counterintuitive. However, as already pointed out in the introduction, subcortical regions including the putamen, striatum and thalamus have been shown to be related to reading previously (). For example, Liu et al. [54] compared reading networks in children and adults and found a greater involvement of subcortical structures in children. According to these author, this “may indicate that children rely on sensorimotor circuits and their connection to cortical linguistic brain regions to maintain sound representations for reading” [54]. The putamen has been suggested to be engaged in the cortical initiation of phonological processing during reading [13].

In the present study, the (ICA-characterized) basal ganglia network itself showed no significant between-group differences. We found only increased functional connectivity between this network and right supramarginal gyrus (posterior division) in untrained FI, which was reversed after training and correlated with WLLP and DRT-1. The left supramarginal gyrus is part of a language network in right-handed subjects. The right one has been found to be involved in empathy [70] and phonological decisions [35]. However, there is evidence for reorganization of this area as a part of a language network after recovery from stroke or operation [38]. It is supposed that the predominant left side of the language network inhibits the right side and after loss of this inhibition the right network could be activated [40, 44]. Whether the increased functional connectivity between right supramarginal gyrus and basal ganglia in untrained FI reflects compensation resulting from daily encountering of linguistic problems as permanent stimuli remains questionable. However, in line with this interpretation this state was normalized after training correlating with improvements in WLLP and DRT-1 scores.

Finally, we investigated functional connectivity of other subcortical areas in terms of their correlation with different networks as a whole to assess the role of large-scale cortico-subcortical interplay in language learning and plasticity. DMN is known to be important for episodic, autobiographical and semantic memory, self-related and social cognitive processes, value-based decision making and emotion regulation [14, 20, 74]. Recent studies showed activation or deactivation of different parts of DMN during different language tasks, emphasizing its role in language understanding and speech production in healthy subjects [6, 67]. We found both reduced and increased functional connectivity between different subcortical areas and different parts of the DMN.

### Effect of literacy training on iFC

Importantly, training-related gains in literacy skills were accompanied by changes in functional connectivity in the FI group that by and large can be characterized in terms of a partial normalization, i.e. the connectivity patterns were changed after training to be closer to the normal reading control group.

For example, the changes of DMN observed in FI were partly reversed after training, which furthermore correlated with improvement of WLLP scores. This shows the relevance of the interaction between DMN and these subcortical areas for language processing.

In addition, the increased connectivity between LFPN and right thalamus in untrained FI was reversed after training, again suggesting a normalizing effect of literacy training on functional connectivity. The role of thalamus and basal ganglia in language processing is complex and has been discussed intensively by Klostermann et al. [45]. The thalamus is a relay area involved in the switching between different cortical areas/networks involved in speech, depending on functional demands. It receives information from one part of a network and communicates it further to areas at different levels of the processing hierarchy, to rapidly and selectively facilitate goal-oriented functions, for example word generation. The differences in connectivity between putamen and thalamus and LFPN may reflect group differences in the interaction/influence of thalamus and basal ganglia with respect to cortical areas needed for language production. Finally, the reduced connectivity between Visual Network-I and right thalamus may show reduced visual and attentional interplay by untrained FI due to language.

### Limitations

As any method, rs-fMRI comes with intrinsic limitations. The situation during rest may be considered as insufficiently controlled [77]. Also resting state activity fails to uncover changes in task strategies or functional compensation that occur only during performance. Analysis of resting state activity therefore should be considered as complementary to functional measures. The next important point is the problem of motion in all fMRI studies. In our study we found no increased motion in both groups and no significant differences between both groups. However, using ICA can further minimize effect of motion on results as described elsewhere in details [16]. Especially it could be shown that ICA methods can reliably identify and account for the artefactual influence of respiratory and cardiovascular signal fluctuation on found networks [4, 7].

## Conclusion

In summary the data presented here show significant changes of functional connectivity in cortical and subcortical areas which partly change after training and correlate with increases in reading skills. Whether any or a number of these areas are candidate for non-invasive brain stimulation like repetitive transcranial magnetic stimulation (rTMS) of transcranial direct current stimulation (tDCS) needs further studies [17, 18, 39, 84]. Here, the question is, if manipulation of the resting state of the brain following stimulation of distinct nodes of networks (e.g., Visual Network-I, DMN or VAN) helps better learning.

## Data Availability

Data will be freely shared by the authors.

## Abbreviations

BGN: Basal Ganglia network
CSF: Cerebrospinal fluid
DAN: Dorsal attention network
DMN: Default mode network
DRT: Diagnostischer Rechtschreibtest
EPI: Echo-planar imaging
FFG: Fusiform gyrus
FI: Functional illiterate
FWHM: Full-width at half maximum
ICA: Independent component analysis
iFC: intrinsic functional connectivity
IPS: Intraparietal sulcus
LFPN: Left-fronto-parietal network
LIFG: Left inferior frontal gyrus
LMTG: Left middle temporal gyrus
MELODIC: Multivariate Exploratory Linear Optimized Decomposition into Independent Components
MPRAGE: Magnetization Prepared Rapid Acquisition Gradient Echo
rsfMRI: resting state functional magnetic resonance imaging
RSN: Resting state network
SBCA: Seed Based Correlation Analysis
STG: Superior temporal gyrus
TFCE: Threshold-free cluster enhancement
VAN: Ventral attention network
VN1: Visual Network-1
WLLP: Würzburger Leise-Leseprobe
WM: White matter
